# Machine-Learning-Enabled Hydrogel Biosensors for Wearable Health Monitoring

**DOI:** 10.3390/gels12050449

**Published:** 2026-05-20

**Authors:** Zhizhou Zhang

**Affiliations:** 1 School of Engineering, The University of Manchester, Manchester M13 9PL, UK; zhizhou.zhang@manchester.ac.uk; 2 Faculty of Engineering, University College London, London WC1E 6BT, UK

**Keywords:** hydrogels, biosensors, machine learning, wearable devices, material design, wearable biosensors, multimodal data fusion, wearable health monitoring

## Abstract

Machine learning (ML) is reshaping the design and deployment of conductive hydrogel biosensors for wearable health monitoring by coupling material chemistry with scalable manufacturing and robust signal analytics. Persistent bottlenecks include hydration stability (dehydration and freezing), data scarcity, device variability, and model transfer across users and environments. Recent advances demonstrate ML-enabled gains across electrochemical, mechanical, optical, and multimodal transduction, improving feature extraction, drift compensation, and generalization in applications spanning electrophysiology, sweat chemistry, and soft tactile sensing. On the material side, polymer informatics and graph-based representations are emerging to predict gel properties and guide composition/structure selection. In analytics, physics-informed models are enhancing impedance and voltammetry interpretation and reliability. Building on these trends, this review outlines standards for dataset curation (metadata on ionic milieu, temperature, humidity history, and mechanical loading) and strategies for cross-user and domain generalization. This review closes with actionable design guidelines for standardization, real-time analytics, and the clinical translation of hydrogel wearables.

## 1. Introduction

Conductive hydrogel-based wearable devices have emerged as promising interfaces for continuous health monitoring and human–machine interaction because they match the softness, water content, and ionic character of tissue, thereby lowering contact impedance and motion artifacts relative to rigid electronics [1,2]. Hydrogels were particularly important because their hydrated polymer networks provided properties like mechanics, ion transport, analyte permeability, bioadhesion, and biocompatibility in one material platform. These features allowed wearable sensors to maintain intimate skin contact, reduce irritation, stabilize electrophysiological and biochemical signals, and support continuous monitoring during movement. Progress in hydrogel chemistry, nanocomposite design, and organohydrogel strategies further extended operation across dry and cold environments and improved comfort during long wear [3,4,5]. Wearable devices generally refer to body worn electronic or bioelectronic systems that continuously collect physiological, biochemical, mechanical, or environmental information during daily activity. These systems have appeared in various forms, including skin mounted patches, flexible electrodes, textile integrated sensors, soft electronic skins, wrist or chest monitors, contact lenses, epidermal microfluidic devices, and implant adjacent interfaces. In health monitoring, these devices were expected to remain comfortable, conformal, stable under motion, and compatible with sweat, skin deformation, and long term contact. At the same time, machine learning matured into a practical engine for material discovery, device optimization, and signal interpretation, which created a timely opportunity to couple data-driven methods with hydrogel bioelectronics across the full stack from ink formulation to on-body analytics [6,7,8,9].

**Figure 1:**
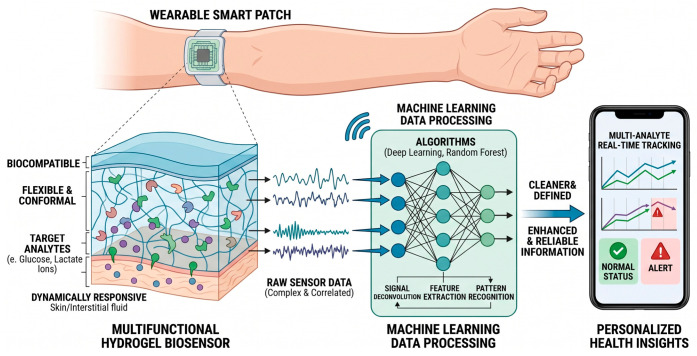
Conceptual illustration of a machine-learning-enabled hydrogel biosensing platform for continuous, personalized wearable health monitoring.

As shown in Figure 1, the schematic outlines the seamless integration of conformal hydrogel–sensor interfaces with advanced data analytics to translate complex physiological signals into actionable medical insights. At the tissue–device interface, a non-invasive wearable smart patch utilizes a biocompatible, flexible hydrogel matrix that establishes intimate skin contact to dynamically detect target analytes—such as glucose, lactate, and essential ions—from interstitial fluid. Because the resulting raw sensor data is often complex and correlated, it is wirelessly transmitted to an algorithmic processing module where machine learning models, including deep learning networks and random forests, perform critical signal deconvolution, selective feature extraction, and pattern recognition. This advanced computational workflow refines the raw data into cleaner, highly reliable clinical information, which is ultimately presented on a user interface (e.g., a smartphone) to enable multi-analyte real-time tracking, immediate health status classification, and predictive personalized health alerts.

Foundational material work established how conductive hydrogels combined stretchable polymer networks with electronic or ionic pathways to deliver stable electrical readout under strain and sweat. Reviews and exemplars documented improvements in adhesion, antifreezing performance, and conductivity using nanofillers, conducting polymers, zwitterionic chemistries, and mixed solvent networks [4,10,11,12]. Interfaces designed as tissue like gels achieved very low skin impedance and conformal contact, which translated into better electroencephalography and electromyography quality during real use [3,13]. In parallel, organic electrochemical transistor platforms and ultrathin gel electrodes pushed density and comfort for wearable or implant adjacent sensing, indicating a route to in-sensor amplification and preprocessing at very low supply voltage [13,14,15].

Manufacturing advances supported translation from laboratory formulations to scalable devices. Photocurable gelatin methacryloyl systems, digital light processing, and stereolithography workflows offered precise patterning of soft networks, while parameter control of initiator, exposure, and layer thickness tuned mechanics and transport for embedded sensors [16,17,18]. Vascular and microfluidic architectures printed inside hydrogels demonstrated how three-dimensional topologies could route fluids, enable chemical sampling, and maintain function under deformation [19]. These capabilities aligned with the needs of skin-mounted or implant-compatible biosensors that required both mechanical fidelity and efficient mass transport.

Concurrently, machine learning reshaped material selection and process optimization. Polymer informatics introduced multitask graph neural networks and self-supervised learning that learned polymer structure property relations directly from graph encodings under data scarcity, enabling rapid screening and inverse design of gel chemistries with targeted elasticity, swelling, and conductivity [20]. In bioprinting and ink optimization, Bayesian optimization and surrogate modeling reduced experimental burden and identified Pareto sets for cell viability and print fidelity across extrusion pressure, speed, and temperature, while new optical assessment tools quantified strand geometry for feedback [21,22]. These methods were well suited to conductive gels whose printability and post-curing determined both mechanical match to tissue and signal stability.

Downstream of fabrication, data-driven analytics improved performance under real-world noise. Reviews across wearable sensing showed that modern classifiers and sequence models improved accuracy and robustness for biosignal interpretation when supplied with better contact quality and richer multimodal features [8]. For electrochemical sensors, learning aided feature extraction from voltammetry and impedance spectra and supported drift resilient concentration estimates in complex media, a requirement for biochemical readouts from sweat or interstitial fluid [7]. Implantable and hydrogel-protected aptamer sensors demonstrated stable continuous molecular measurements in vivo, establishing high-value targets for uncertainty-aware signal processing and control [14].

Despite this momentum, important challenges remained. Hydrogel devices still faced dehydration, freezing, and biofouling that altered impedance and baseline stability during prolonged wear, although organohydrogels and antifreezing chemistries mitigated these effects [4,23]. Model transfer across users, body sites, and sessions remained difficult because dataset sizes were modest and covariates varied, motivating standardized protocols, domain adaptation, and calibrated uncertainty in deployment [8]. Integration at the system level required low-power front ends and reliable on-board inference, where organic electrochemical transistor arrays and conformable ultrathin electrodes suggested practical paths forward [13]. Finally, emerging modalities such as electrical impedance tomography-based skins and triboelectric impedance tomography patches broadened spatial sensing but introduced new inverse problems where learning- and physics-based priors needed to work together [24].

This review contributes a materials-to-analytics perspective on machine learning for conductive hydrogel wearables. We summarize the background on conductive and organohydrogel platforms that enabled stable biointerfaces [11,12]. We survey prior work that applies graph models, self-supervised learning, and Bayesian optimization to polymer design, ink formulation, and three-dimensional printing relevant to gel-based devices [21,22]. We examine current challenges in dataset curation, generalization, hardware software co-design, and environmental stability that constrain translation [13,24]. We then outline how these ingredients can be combined into closed-loop workflows for discovery, manufacturing, and robust on-body sensing, with emphasis on opportunities where data-efficient learning and uncertainty reporting would accelerate safe and reliable deployment.

## 2. Hydrogel-Based Biosensors

Soft biosensors based on hydrogels combine the virtues of soft-matter mechanics with biochemical recognition to yield sensor interfaces that more closely match biological tissues. Hydrogels, being water-rich, flexible, and biocompatible, mitigate the mechanical mismatch between stiff sensors and soft tissues, thus reducing delamination, tissue damage, and motion artifacts. For instance, hydrogel interfaces have been demonstrated to reduce motion-induced artifacts in electrophysiological recording by conformally coupling with curved surfaces [25]. In recent years, the confluence of advances in hydrogel chemistry, conductive fillers, stretchable electronics, and biofunctionalization have driven a surge of interest in soft hydrogel biosensors as next-generation platforms. Reviews in the broader domain of hydrogel bioelectronics have highlighted trends in conductive hydrogels, implantable soft bioelectronics, and interfacing strategies [26,27]. Hydrogels are three-dimensional hydrophilic polymer networks that can absorb and retain large amounts of water while maintaining a solid like structure, which gives them tissue like softness, high permeability, and good biocompatibility [28,29]. They can be prepared through physical crosslinking, chemical crosslinking, photopolymerization, ionic coordination, freeze–thaw cycling, supramolecular assembly, or hybrid strategies that combine covalent and reversible interactions [18,30]. In wearable biosensors, conductive or functional hydrogels are often formed by incorporating ionic salts, conducting polymers, carbon nanomaterials, metal nanoparticles, enzymes, antibodies, aptamers, fluorescent probes, or pH-responsive molecules into the polymer network [31,32]. These preparation strategies allow hydrogels to be tailored for different sensing mechanisms, including electrochemical detection, optical readout, strain or pressure sensing, temperature monitoring, and multimodal signal acquisition [33]. Because of their softness, stretchability, adhesion, water-rich transport pathways, and low-impedance contact with biological tissues, hydrogels have been widely applied in wearable sweat sensors, electrophysiological electrodes, motion sensors, wound-monitoring devices, implantable biointerfaces, and human–machine interaction systems.

### 2.1. Hydrogel Design Criteria and Strategies

A soft hydrogel biosensor had to satisfy several coupled requirements, including mechanical softness and stretchability, stable electrical connection to electrodes or signal transducers, biocompatibility, and efficient analyte transport within the hydrated polymer network. Matching the modulus of the target tissue was often used as a first selection rule, and hydrogels with moduli in the range of low kPa to tens of kPa were frequently selected for epidermal and biointerface applications [26]. The material choice then depended on the target sensing task. Conductive nanocomposite hydrogels that contained carbon nanotubes, graphene, metallic nanoparticles, conducting polymers, or ionic liquids were selected when stable electrical readout, strain sensitivity, and low-impedance skin contact were required. Double network and interpenetrating network hydrogels were selected when large deformation, toughness, and repeated mechanical loading were important, because one network provided strength, while the other preserved elasticity [34]. Organohydrogels and salt-rich ionic hydrogels were selected for long-term or outdoor wearable devices because they reduced dehydration and freezing while maintaining ionic conductivity. Adhesive hydrogels based on catechol, ionic, hydrogen bonding, or covalent interactions were selected when stable skin attachment and reduced motion artifacts were needed. Stimuli-responsive hydrogels were selected for chemical or optical biosensing because their swelling, fluorescence, color, or conductivity changed in response to pH, temperature, glucose, ions, or biomolecular binding. Semiconducting hydrogel systems were selected when soft mixed ionic and electronic transport was required for bioelectronic interfaces [35]. In this way, each hydrogel material in the reviewed examples was chosen not only for softness, but also for a specific combination of conductivity, stretchability, adhesion, environmental stability, analyte permeability, and transduction compatibility.

Table 1 compiles publicly reported hydrogel datasets, covering printability, mechanics, adhesion, and gelation, and summarizes dataset scope, descriptor types with SI units, and bibliographic sources. The datasets summarized in Table 1 were compiled from publicly reported hydrogel studies and data repositories that provided explicit links between formulation, structure, processing conditions, and measurable material properties. We selected datasets that contained machine-readable inputs, such as monomer composition, molecular descriptors, rheological parameters, solvent conditions, crosslinking variables, or printing parameters, together with outputs relevant to wearable hydrogel biosensors, including gelation outcome, modulus, viscosity, swelling, adhesion, yield stress, and printability. This compilation strategy was used because machine learning models required paired input–output data rather than isolated material descriptions. The selected datasets covered both classification tasks, such as gelator-versus-non-gelator or printable-versus-non-printable systems, and regression tasks, such as predicting modulus, adhesion strength, viscosity, or yield stress. Therefore, the compiled data was not intended to be an exhaustive catalogue of all hydrogel studies. Instead, it was used to illustrate how curated hydrogel datasets could support descriptor selection, model training, benchmarking, and data-driven formulation design for wearable biosensing materials.

Conductive fillers such as carbon nanotubes, graphene, metallic nanoparticles, and ionic liquids are widely used to impart electrical responsiveness. Roy et al. reviewed the role of conducting nanocomposite hydrogels in wearable biomonitoring, emphasizing design guidelines for sensitivity, stability, self-healing, and multifunctionality [32]. Meanwhile, Shin et al. [31] summarized advances in conductive hydrogels for biomedical soft devices, focusing on integration into soft sensors and stimulation platforms. Another design dimension was dynamic or reversible bonding (e.g., supramolecular crosslinks, hydrogen bonds, ionic crosslinks) to allow for self-healing and durability under strain cycles.

Beyond conductivity, the hydrogel matrix itself could serve as a responsive medium: swelling/deswelling, volume changes, and network rearrangements could transduce analyte concentration into mechanical or optical signals. Hydrogels responsive to pH, temperature, ionic strength, or light have been used as direct transducers, sometimes coupled with embedded optical or fluorescent probes [33]. Smart hydrogel sensors that respond to glucose or specific biomolecules have been demonstrated by embedding enzyme or aptamer elements in the hydrogel network [43].

**Figure 2:**
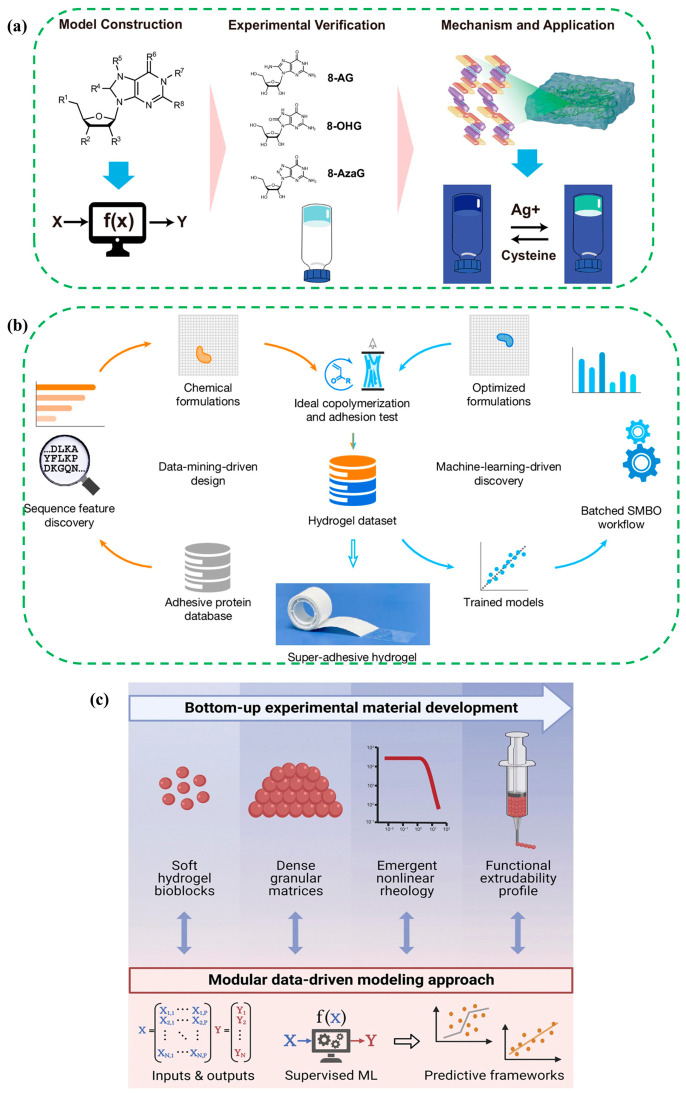
(**a**) An optimal model was constructed for nucleoside derivatives’ hydrogel-forming ability prediction, and the hydrogel-forming ability was experimentally verified. The hydrogel was applied in rapid visual detection of Ag^+^ and cysteine [38]. (**b**) Conceptual scheme of the proposed approach integrating data mining (DM), experimentation and machine learning (ML) to design high-performance adhesive hydrogels [40]. (**c**) Modular approach integrates data-driven modeling with experimentation to map the input–output relationships at each stage of soft granular matrix development [45]. Panels adapted with permission from: (**a**), ref. [38], Nature, CCBY 4.0; (**b**), ref. [40], Nature, CCBY 4.0; (**c**) ref. [45], Cell Press.

In the design of implantable or bioelectronic systems, additional functional traits such as biodegradability, bioadhesiveness, injectability, and self-healing are often engineered into the hydrogel networks [44]. In Figure 2a, the central design inspiration was that predictive features with the largest weights related to hydrogen bonding capacity, molecular polarity, and lipid solubility, which guided selection of nucleoside derivatives expected to assemble through directional interactions [38]. The optimal logistic regression model trained on 71 compounds with 4175 descriptors (reduced to 24 task critical variables) delivered a test accuracy 0.71. Experimental validation then identified two guanosine derivatives that formed cation-independent hydrogels through dynamic borate diester bonds, favored anti-glycosidic conformations that promoted G ribbon assembly rather than G quartet stacking, showed storage modulus greater than loss modulus across the measured angular frequency window. Combining descriptor-based machine learning with supramolecular theory enabled targeted discovery of long lived, self-healing, cation-independent nucleoside hydrogels and suggested practical sensing utility where rhodamine 123 fluorescence was restored by Ag^+^ at 10 µmol L^−1^ within 10 min and quenched again by cysteine at 10 µmol L^−1^ within 10 min, providing a clear material-to-function pathway for future hydrogel design.

Figure 2b depicts an end-to-end pipeline in which data mining of adhesive protein databases informed polymer formulation, laboratory synthesis and iterative machine learning to realize super-adhesive underwater hydrogels [40]. Adhesive protein sequences from thousands of organisms were coarse-grained into six functional classes and converted into relative composition descriptors that could be reproduced statistically by ideal random copolymerization, a strategy justified by the Mayo Lewis model for sequence statistics and by near-unity reactivity ratios that suppressed composition drift during polymerization. The initial dataset comprised 180 bioinspired hydrogels prepared in dimethyl sulfoxide and screened under a standard tack protocol on glass in normal saline at 0.154 M sodium chloride with a loading force of 10 N and a contact time of 10 s, yielding 16 gels with adhesive strength greater than 100 kPa, 83 gels above 46 kPa and a best data-mining-driven specimen at 147 kPa. Gaussian process and random forest regressors were trained on the six-dimensional composition vector and embedded in a sequential model-based optimization workflow with expected improvement to propose new compositions, which expanded the dataset to 341 hydrogels and delivered a formulation that exceeded 1 MPa underwater adhesive strength on glass, an improvement of an order of magnitude over typical underwater gel adhesives reported previously.

In Figure 2c, the key finding and inspiration for hydrogel material design was that coupling ionic control of alginate swelling and stiffness with compaction governed by jamming allowed for machine-learning-guided selection of the triad of alginate concentration, calcium-to-sodium ion balance, and solid volume fraction to set storage modulus from about 0.1 Pa to about 11 kPa to tune yield stress over more than three orders of magnitude, and to delineate injectability boundaries for printable and injectable hydrogels. Rheology showed storage modulus above loss modulus in the linear regime followed by viscoplastic yielding. Injectability classifiers reached an accuracy of 0.885 and an area under the receiver operating characteristic curve of 0.955, and force prediction had a median absolute error of 0.617 N with higher complex-modulus-increasing force and smaller nozzles inducing end-of-stroke-force growth. Ionically crosslinked alginate droplets were formed by air-assisted extrusion into an aqueous calcium chloride bath, and data-driven classifiers predicted successful particle formation with an accuracy of 0.912, showing that a larger nozzle diameter from 30 µm to 150 µm and larger pressure-to-nozzle distance from 1 mm to 30 mm produced more than 60% larger bioblocks, grounded in theories of jammed matter, percolation-like transitions in contact networks, and supervised ensemble learning with nested and grouped cross-validation plus permutation importance.

To make the material selection logic clearer across the case studies, the reviewed hydrogel examples could be grouped by their dominant design purpose. Nucleoside-derivative hydrogels were selected because their hydrogen bonding capacity, molecular polarity, and lipid solubility promoted supramolecular assembly, which made them suitable for predictive gelation and Ag^+^ or cysteine sensing [38]. Bioinspired adhesive hydrogels were selected because protein-derived compositional descriptors guided strong underwater adhesion, which was useful for stable wet interfaces and bioadhesive wearable systems [40]. Alginate granular hydrogels were selected because calcium mediated ionic crosslinking and jamming controlled stiffness, yield stress, injectability, and printability, which made them useful for printable and injectable soft matrices [45]. Antifreezing ionic hydrogels were selected because their ion-rich or mixed-solvent networks preserved conductivity and stretchability under cold or dry conditions. Gelatin-based conductive hydrogels were selected because their soft, hydrated, and conductive network supported subtle strain detection. Carbon-nanotube-containing hydrogels were selected because the conductive filler network provided high electrical conductivity and repeatable skin adhesion. These examples showed that hydrogel selection was application-driven, and the chosen material properties directly determined the sensing mode, operating environment, and signal stability.

### 2.2. Sensing Modalities and Transduction Mechanisms

Soft hydrogel biosensors had been implemented using multiple transduction modalities, often categorized into: (i) electrochemical, (ii) optical (fluorescence, absorbance, colorimetry), (iii) mechanical/physicomechanical (volume or strain changes), and (iv) hybrid or multimodal approaches.

In this chapter, we recapitulate the sensing modalities relevant to soft biosensors constructed from hydrogels. Hydrogels are three-dimensional polymer networks that can absorb and retain large amounts of water, giving them a soft and flexible nature that mimics biological tissues [46]. This intrinsic biocompatibility minimizes inflammatory responses and enhances the long-term stability of implanted biosensors. The porous structure of hydrogels provides a high surface area for the immobilization of biorecognition elements such as enzymes [47], antibodies [48], and nucleic acids [49], leading to improved sensitivity and faster response times. Furthermore, the chemical composition of hydrogels can be tailored to respond to specific stimuli, such as pH, temperature, or the presence of specific biomolecules, enabling the development of smart biosensors with built-in signal transduction mechanisms [50].

**Figure 3:**
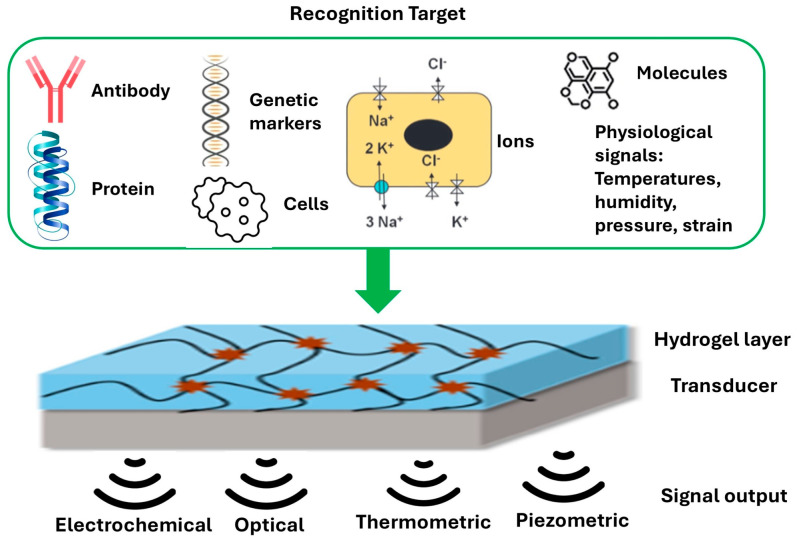
Sensing modalities and transduction mechanisms of hydrogel-based biosensors.

Hydrogel biosensors couple molecular recognition in a soft, water-rich network to physical signal conversion by an underlying transducer as shown in Figure 3. In electrochemical mode [15], target binding or reactions in the gel alter ionic conductivity, charge transfer, and redox currents, which the electrode reads as changes in impedance, potential, or faradaic current. In optical mode [51], recognition sites modulate absorbance, fluorescence, or refractive index within the hydrated network, and the photodetector captures the resulting intensity or phase shift. In thermometric mode [52], exothermic or endothermic events and thermoresponsive swelling change local heat transport and resistance, which the thermistor or microheater bridge converts into a temperature-dependent signal. In piezometric mode [53], mechanical inputs such as pressure, strain, and shear deform the polymer network and percolated pathways, which produces measurable changes in resistance, capacitance, or resonant frequency at the transducer. Together, these modalities translate biochemical and biophysical cues into robust electrical or optical outputs while preserving intimate tissue contact and mass transport in the hydrogel layer.

Electrochemical biosensors represent one of the most promising applications of hydrogel technology. In these devices, hydrogels serve as a versatile platform for immobilizing enzymes and other biorecognition molecules onto electrode surfaces [54]. The high water content and porous structure of the hydrogel facilitate the transport of analytes and reaction products, leading to enhanced electrochemical signals. [55] For example, researchers developed a hydrogel-based electrochemical biosensor for the sensitive detection of the cancer biomarker HER2 in human serum [56]. The hydrogel provided a biocompatible environment for the immobilized antibodies and effectively prevented nonspecific protein adsorption, resulting in a highly sensitive and selective biosensor.

Table 2 compares the role of hydrogels, signal outputs, representative wearable applications, and key advantages across electrochemical, optical, mechanical, and multimodal systems. This summary helps readers quickly distinguish how different hydrogel platforms converted biochemical or biophysical changes into measurable signals.

Hydrogels found widespread use in the development of optical biosensors. These devices utilize changes in optical properties, such as color or fluorescence, to detect the presence of target analytes. For instance, scientists created a pH sensor based on a hydrogel optical fiber, where a pH sensitive fluorescent indicator was doped into the core of the fiber [51]. The sensor exhibited a linear response to pH changes in the physiological range, demonstrating its potential for in vivo pH monitoring. Another innovative approach involved the use of photonic crystal hydrogels, which change color in response to glucose concentration, offering a visual and noninvasive method for glucose monitoring [64].

The unique mechanical properties of hydrogels, such as their softness, stretchability, and self-healing capabilities, make them particularly well suited for the fabrication of wearable biosensors [65]. These devices can conform to the skin and provide continuous monitoring of physiological parameters without causing discomfort or irritation. For example, a wearable multimodal touch sensor was developed using a stretchable and self-healable conductive hydrogel [66]. The sensor could simultaneously monitor touch and temperature, highlighting the potential of hydrogel-based soft electronics for applications in smart prosthetics and human–machine interfaces.

Multimodal hydrogel biosensors synergistically combine two or more transduction mechanisms to enhance sensitivity, reliability, and resilience against interfering signals. Sun et al. [67] reported a hydrogel interface capable of simultaneously sensing motion, temperature, and urea via combined electrical and physicochemical transduction, functioning as a wearable and implantable bidirectional interface for both biosensing and stimulation. A flexible hydrogel sensing patch recently realized a tri-mode integration of pressure, temperature, and proximity sensing with minimal crosstalk, using distinct transduction pathways for each modality [63]. In the domain of respiratory monitoring, a multimodal hydrogel sensor combined resistive (electrical) and capacitive/pressure sensing in a single device to accurately track breathing dynamics and diagnose conditions such as obstructive sleep apnea [63]. More recently, multimodal and flexible hydrogel sensors have been developed specifically for respiratory health, integrating multiple sensing channels in a deformable hydrogel architecture to deliver complementary physiological information [68]. In human–machine interaction (HMI), a hydrogel sensor array concurrently acquired electromyographic (EMG) and force myographic (FMG) signals, and the dual modalities improved gesture recognition accuracy in rehabilitation tasks versus single-channel systems [69]. In soft robotics, hydrogels combining conductive and optical properties enabled optical (transparency or light modulation) and resistive sensing within the same element to discern both deformation state and ambient temperature [70]. Finally, anisotropic deformation sensing hydrogels with multiple directional mechanical responses (e.g., tensile, compressive, shear) offered multimodal mechanical outputs that could decode complex motion patterns, emulating aspects of neural proprioception [71].

The applications of hydrogel-based biosensors are vast and continue to expand. In the field of healthcare, hydrogel biosensors developed for a wide range of applications, including continuous glucose monitoring [72] for diabetes management, point of care testing for infectious diseases [73], and the early detection of cancer biomarkers [74]. Their biocompatibility also makes them ideal candidates for in vivo monitoring of physiological parameters, such as pH [75], bacterial infections [76], and ion concentrations [77]. Beyond healthcare, hydrogel biosensors are being explored for applications in environmental monitoring, food safety, and bioprocess control [78].

Table 3 shows that machine learning has improved biosensor analysis across electrophysiological, biochemical, mechanical, and material-design tasks. However, direct comparison remains difficult because studies use different datasets, validation schemes, and performance metrics. Large-scale ECG models report high AUROC values, whereas hydrogel-specific studies usually rely on smaller datasets and controlled conditions. Multimodal sensing improves accuracy, but subject-wise validation, long-term wear testing, uncertainty calibration, and cross-device transfer remain insufficiently reported.

## 3. Machine Learning Methods for Hydrogel Biosensors

### 3.1. Descriptors and Features of Hydrogel Materials and Sensing

Quantitative descriptors of the swollen polymer network govern transport, mechanics, and interfacial behavior and therefore define what a sensing hydrogel can detect and how it transduces signals [83,84,85,86,87,88,89,90,91,92,93,94,95]. Network parameters such as polymer volume fraction, crosslink density, and mesh size were linked through Flory–Rehner and Peppas–Merrill formalisms and related extensions, which expressed the elastic free energy and osmotic mixing terms and yielded estimates of average chain length between crosslinks and mesh size in nanometer units [96,97,98]. In practice, soft biointerfaces targeted an elastic modulus between 0.1 kPa and 10 kPa to match neural and other soft tissues and to limit motion artifacts during wear [99,100]. Interfacial descriptors such as adhesion energy reached values on the order of 10^3^ J/m^2^ for tough hydrogel elastomer interfaces, which supported stable skin contact in long measurements [101], and molecular strategies for catechol, ionic, and covalent bonding allowed for design control over strength and reversibility [102].

Transport descriptors controlled analyte delivery to recognition motifs. Diffusion coefficients for small solutes in hydrogels typically decreased relative to water by 5% to 20% for saccharides and by about 50% for macromolecules such as vitamin B12, which corresponded to D values near 3 × 10^−10^ m^2^/s to 6 × 10^−10^ m^2^/s for small molecules in hydrated networks at room temperature [103,104]. Mesh size theory explained these reductions by steric obstruction and hydrodynamic drag within the swollen network and provided closed-form expressions that connected diffusion to polymer volume fraction and Kuhn length [98]. For ionic and mixed ionic electronic hydrogels, bulk descriptors included ionic conductivity σ and Donnan potential. State-of-the-art skin interfacing gels reported σ in the 0.1 S/m to 10 S/m range, sufficient to minimize series resistance at the skin interface and to stabilize potentials during motion [105,106], while porous and nanomesh organic electrochemical transistor platforms leveraged high volumetric capacitance to amplify low level ionic signals at sub-1 V supply [107] and benefited from hydrogel electrolytes with matched mechanics and high water content [108].

Sensing features extracted from the hydrogel transducer stack summarized the relevant physics for data analysis and machine learning. For electrochemical impedance spectroscopy, equivalent circuit parameters such as solution resistance, double layer capacitance, charge transfer resistance, and the Warburg diffusion element served as compact features and mapped to interfacial chemistry and transport [109]. Data-driven impedance analysis further employed distribution of relaxation times or circuit discovery to reduce model error relative to the classic Randles circuit, with recent studies reporting root mean square fitting errors below 5% for 10^2^ to 10^3^ frequency points between 1 Hz and 10^5^ Hz [110]. At the tissue interface, contact impedance acted as a critical feature for electrophysiology quality. Wet or semidry electrodes with hydrogel contact often achieved 2 kΩ to 5 kΩ at 10 Hz, whereas dry contacts frequently exceeded 20 kΩ under similar conditions, which increased noise and trial counts needed for reliable signals [111].

Optical and mechanical transduction provided additional feature sets. In optical readouts, absorbance or fluorescence intensity and lifetime changes were paired with refractive index shifts to quantify binding events in the hydrogel. These metrics were conveniently summarized as sensitivity, limit of detection, and response time that were governed by diffusion and mesh size [103,104]. In piezometric and capacitive modes, resistance change, capacitance change, and resonance frequency shift captured deformation of percolated paths and effective permittivity in the hydrated network, which were modulated by crosslink density and solvent content described above [101]. Collectively, these descriptors connected network chemistry to transport and interface physics, and the derived sensing features provided compact targets for model training and design optimization that reduced prediction error while guiding hydrogel selection for specific analytes and operating environments.

### 3.2. Machine Learning Benchmarking for Hydrogel Biosensors

Supervised benchmarking for hydrogel biosensors was strongest when tasks were separated into time-series classification for biosignals and regression for physicochemical quantities, with principled dataset splits and calibration. Subject-wise folds prevented identity leakage in electroencephalography or electrocardiography classification, while stratified splits by analyte level and matrix composition limited covariate shift in concentration or impedance regression. Core metrics included accuracy, F1 score, area under the receiver operating characteristic curve, mean absolute error, root mean square error, and median absolute percentage error with reliability diagrams for probability calibration. Scale references from clinical corpora set expectations for generalization: a deep network trained on 91,232 single-lead electrocardiography records achieved an area under the receiver operating characteristic curve equal to 0.97 across 12 rhythm classes [79], and an extended Temple University Hospital electroencephalography resource added 15,300 automatically derived pathology labels for supervised evaluation [112]. Hydrogel electrodes influenced these outcomes by lowering contact impedance and stabilizing skin–electrode coupling. A conductive and adhesive layered hydrogel maintained high-quality electroencephalography for up to 10 h with low contact impedance [13], and a tough semi-dry hydrogel variant supported repeatable long-duration recordings [113]. Quantitative analyses showed wet electrodes delivered contact impedance below 800 Ω at 10^2^ to 10^3^ Hz, semidry around 1.5 kΩ, and dry near 80 kΩ, underscoring the material link to classifier headroom [111,114]. Benchmark design benefited from task-specific splits and calibration, and the combination of validated scales and low contact impedance hydrogel interfaces provided realistic targets for accuracy above 90% and stable probability calibration on datasets of 10^3^ to 10^5^ labeled windows.

Electrochemical impedance spectroscopy offered physics-grounded benchmarks that mapped spectra to equivalent circuit parameters under constraints from Kramers–Kronig relations and diffusion controlled Warburg behavior [109]. Reviews of machine learning in electrochemical biosensing framed data preprocessing, model selection, and uncertainty for classification of circuit forms and regression of parameters [7]. Deep learners classified equivalent circuit families and regressed parameters from spectra sampled over 0.1 to 100 kHz with held-out errors at or below a few percentage points [115]. Resource-constrained approaches executed fitting of simplified Randles circuits on microcontrollers with memory on the order of kilobytes and achieved measurement accuracy between 1% and 3% for spectra over 0.1 to 10 kHz, demonstrating feasibility for embedded hydrogel sensors [116]. As an environmental analog for on-body drift, supervised models trained on 38 d of plant bioimpedance achieved consistent generalization across 10^2^ to 10^6^ Hz while tracking circuit parameter trajectories during stress [117]. At the biochemical interface, reviews of microfluidic machine learning triads and analytical work on electrochemical aptamer sensors identified drift sources such as surface fouling and nonspecific adsorption and recommended referencing and filtering strategies for stable continuous operation [117,118]. For in-sensor amplification and preprocessing under low supply voltage, internal ion gated organic electrochemical transistors operated stably in physiologic electrolytes for more than 1 y and informed latency and memory budgets for on device supervised inference [118,119]. Benchmarkable targets for hydrogel electrochemical sensing included circuit parameter regression root mean square error below 5% over 0.1 to 100 kHz and concentration regression median absolute percentage error near 10% when drift compensation, physics-constrained modeling, and edge-efficient learners were combined.

Materials and manufacturability connected hydrogel design to supervised performance through polymer informatics and print process control. Multitask graph neural networks and self-supervised graph neural networks improved prediction of polymer elastic modulus, glass transition temperature, ionic conductivity, and swelling ratio from repeat unit graphs. Reported mean absolute error reductions of about 10 to 30% accelerated screening of hydrogel formulations that target low modulus and high ionic transport for bioelectrodes [120]. Reviews of electrochemical impedance spectroscopy in biological media supplied guidance on frequency selection and feature construction for supervised learners, while portable impedance platforms with interdigitated electrodes defined realistic sampling densities and noise floors for training [121,122]. At the device level, fully screen-printed gentle-to-skin wet electrodes captured clinical grade electrocardiography with compact wireless readouts [120], and fully textile polymer electrodes from the biomedical engineering literature offered ambulatory reference baselines with low irritation [123]. In fabrication, neural network Bayesian optimization and extrusion speed flow models identified Pareto optimal printing conditions in tens of experiments and reduced line width error by roughly 20 to 40%, which lowered variance in contact impedance and improved the reproducibility of downstream supervised models [21]. Data informed polymer selection and process optimization supported benchmark goals such as accuracy above 0.90 for posture or event detection, root mean square error below 5% for equivalent circuit regression, and median absolute percentage error around 10% for analyte regression on datasets of 10^3^ to 10^5^ spectra or windows, with reproducibility anchored by standardized reporting of frequency spans, contact impedance in Ω at specified frequencies, and device aging.

**Figure 4:**
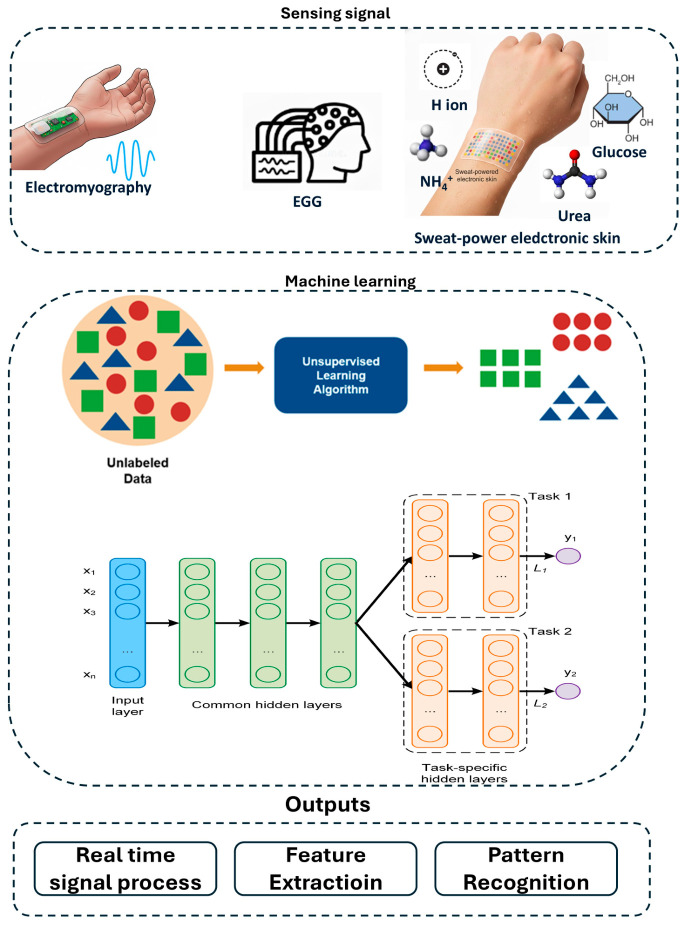
Machine learning workflow linking hydrogel biosensor signals to multitask outputs.

Figure 4 illustrates how hydrogel-based wearables acquired electrophysiology and sweat chemistry signals (electromyography, electroencephalography, H^+^, NH_4_^+^, glucose, urea), applied unsupervised learning to organize unlabeled data, and then used a shared backbone with task specific heads for supervised inference to enable real-time processing, feature extraction, and pattern recognition.

From an algorithmic perspective, different machine learning methods showed distinct strengths in performance, robustness, and applicability for hydrogel biosensors. Classical supervised models such as support vector machines, random forests, and gradient boosting were suitable for small or medium-sized datasets with engineered features, including impedance parameters, peak currents, gauge factors, and spectral intensities. These models were relatively interpretable and robust under limited data, but their performance depended strongly on feature quality. Deep learning models, including convolutional neural networks, recurrent neural networks, and transformer-based architectures, were more effective for high-dimensional waveforms, spectra, images, and multimodal time series because they could learn latent features directly from raw signals. However, they usually required larger datasets, careful regularization, and cross-user validation to avoid overfitting. Bayesian optimization and Gaussian process models were more applicable to hydrogel formulation and printing optimization, where experiments were expensive and uncertainty-guided candidate selection was valuable. Graph neural networks and self-supervised polymer models were more suitable for material informatics because they encoded molecular structure or polymer repeat units and supported property prediction under data scarcity. Physics-informed and hybrid models improved robustness by embedding electrochemical, mechanical, or transport constraints into learning, which reduced unphysical predictions and improved generalization under drift. Transfer learning and domain adaptation were especially important for wearable deployment because sensor responses varied across users, body sites, sessions, and environments. Therefore, algorithm choice should depend on biosensor modality, dataset size, feature type, interpretability requirement, and deployment condition rather than on accuracy alone.

To provide a more analytical comparison, Table 4 summarizes how different machine learning algorithms matched different hydrogel biosensor data types and deployment needs. The comparison showed that algorithm selection should not be based only on prediction accuracy. Classical models such as SVM, random forests, and gradient boosting were more suitable for small datasets with engineered features, whereas deep learning models were more useful for high-dimensional waveforms, spectra, and multimodal time series. Bayesian optimization and graph neural networks were more relevant to hydrogel formulation and material informatics, while physics informed models, transfer learning, and multimodal fusion were important for improving robustness under drift, user variability, and real wearing conditions. Therefore, the critical point was that each algorithm family had a different balance between performance, interpretability, data requirement, and deployment robustness.

Earlier machine learning studies in biosensors had often failed when training data did not represent the sensing conditions seen during deployment. Deep learning achieved high performance for waveform tasks when the data scale was large. For example, a network trained on 91,232 single-lead electrocardiogram records from 53,549 patients reached an average area under the receiver operating characteristic curve of 0.97 for 12 rhythm classes [79]. Yet, this level of performance depended on large labeled datasets, expert annotation, and stable sensor quality. Electroencephalogram studies showed the opposite limitation. Earlier abnormal electroencephalogram models had relied on about 3000 labeled recordings, and a later expansion added 15,300 automatically labeled recordings, with a balanced subset of 8879 recordings [112]. This showed that small datasets limited generalization, while automatic labels introduced label noise. In sweat and electrochemical biosensors, failures came from drift, fouling, sweat rate changes, temperature variation, and weak transfer from controlled experiments to daily use. Multiparametric sweat modeling used heart rate, sweat rate, and sweat lactate to estimate blood lactate, but error still depended on subject physiology and exercise state [131]. Method theories explained these failures. Bias and variance theory showed that small datasets increased variance. Domain adaptation theory showed that user, body site, and session shifts changed the input distribution. Calibration theory showed that fixed calibration curves failed when baseline drift and matrix effects changed the sensor response. Key finding was that optimal performance in biosensor machine learning required enough labeled data, subject wise validation, drift aware calibration, and uncertainty reporting, rather than a high accuracy value from a single controlled dataset.

Optimal machine learning performance had to be defined differently for different biosensor tasks. For classification of electrocardiogram, electroencephalogram, and electromyogram signals, the best target was high sensitivity, high specificity, calibrated probability, and stable cross-user accuracy. Deep convolutional models were suitable when datasets reached tens of thousands of windows, while support vector machine, random forest, and gradient boosting models were more reliable for smaller datasets with engineered features such as impedance, peak current, or spectral intensity. For regression tasks such as electrochemical concentration estimation, the optimal target was low root mean square error, low mean absolute error, and stable error after drift correction. For material design, optimal performance meant fewer experiments and reliable structure property prediction. In alginate granular hydrogel matrices, data-driven models predicted injectability with an accuracy of 0.885 and an area under the receiver operating characteristic curve of 0.955. Force prediction reached a median absolute error of 0.617 N, and particle formation classification reached an accuracy of 0.912 [45]. In polymer informatics, graph neural networks reduced manual feature engineering by learning from repeat unit graphs, while self-supervised graph neural networks reduced root mean square error by 28.39 percent for electron affinity and 19.09 percent for ionization potential under scarce data [9,20]. Design theory suggested that the best results came from matching the model to the task. Bayesian optimization worked best when experiments were expensive. Graph neural networks worked best when molecular structure controlled properties. Physics-informed models worked best when impedance, diffusion, or mechanics constrained the solution space. A key finding was that optimal performance was achieved by task matched model selection, physically meaningful features, standardized splits, uncertainty-guided experiments, and validation across users, sessions, and operating environments.

## 4. Machine Learning Applications for Hydrogel Biosensors

Traditional hydrogel biosensors usually relied on empirical material selection, fixed calibration curves, single or limited sensing channels, and manually defined signal features. This strategy was effective for demonstrating proof-of-concept devices, but it often struggled when hydrogel properties changed with dehydration, swelling, temperature variation, mechanical deformation, biofouling, or user-to-user differences. In contrast, machine learning enhanced hydrogel biosensors used data-driven models to connect formulation, processing, device structure, and sensing outputs across larger variable spaces. During material design, machine learning helped screen hydrogel compositions, predict gelation, modulus, conductivity, adhesion, and printability, and reduced trial-and-error experiments. During device operation, machine learning supported drift compensation, noise filtering, feature extraction, multimodal data fusion, and cross-user adaptation. Therefore, the key advantage of applying machine learning was not simply higher prediction accuracy. It also improved design efficiency, robustness under real wearing conditions, and applicability across different sensing tasks, including electrophysiology, sweat analysis, strain monitoring, and multimodal health assessment. Artificial intelligence has become a core engine for the full stack of hydrogel biosensing, from ink design and print process control to device calibration and interpretation of multi modal signals. Recent reviews across soft bioelectronics and wearable analytics converge on the same message. Learning-based methods provide faster iteration, improved selectivity, and resilience to noise while preserving soft matter advantages such as tissue matching and high water content [8,132,133,134].

### 4.1. Material and Formulation Informatics

Material informatics provided a systematic framework for applying machine learning to hydrogel design by linking material composition, molecular structure, processing conditions, and measured properties. In a typical workflow, hydrogel formulations were first converted into machine-readable descriptors, such as monomer type, crosslinker concentration, solvent composition, ionic strength, filler content, polymer topology, molecular fingerprints, BigSMILES strings, or graph-based repeat unit representations. These descriptors were then paired with target properties, including gelation outcome, elastic modulus, swelling ratio, conductivity, adhesion strength, viscosity, yield stress, printability, biocompatibility, and sensing performance. Supervised learning models were used to predict these properties, while uncertainty-aware models helped identify where predictions were reliable and where new experiments were needed. After training, the models supported forward screening of candidate hydrogels and inverse design of new formulations that met multiple wearable device requirements, such as low modulus, high ionic conductivity, strong adhesion, environmental stability, and feasible processing. Therefore, machine learning did not replace polymer physics or hydrogel chemistry, but helped organize experimental data, reveal structure property relationships, and guide efficient discovery of hydrogel materials for wearable biosensors. Machine learning now supports forward prediction and inverse design of hydrogel chemistry and structure. For example, Li et al. [38] trained a model that predicts the gel forming ability of nucleoside derivatives and then uses these predictions to narrow synthesis space, which shortens the cycle from candidate enumeration to gel confirmation. In polymer informatics, self-supervised [20] and graph-based models [9] encode repeat units and connectivity to predict properties under data scarcity, which is common for bespoke hydrogel systems.

Inverse design of hydrogel chemistry and structure uses a closed loop where a forward surrogate learns mappings from composition or sequence to gelation, mechanics, and transport, and an optimizer or generator inverts that map under synthesizability and safety constraints. In practice, this combines learned structure representations such as BigSMILES and graph encodings with uncertainty-aware objectives and feasibility priors to propose candidates that are both synthesizable and likely to meet multi-objective targets [9,135,136,137]. For supramolecular systems, human-in-the-loop campaigns that fuse coarse-grained simulation with classifiers have already mapped 160,000 tetrapeptides and achieved an 87.1% gelation hit rate among the top eight thousand sequences, demonstrating how iterative learning sharply improves discovery yield [138]. Small-molecule gelators based on nucleosides have likewise moved from intuition to predictive selection, where a descriptor model trained on seventy one derivatives reached 71% accuracy internally and achieved 83.3% external verification when ranking new candidates, including the discovery of cation-independent gels that were then validated experimentally [38]. For polymeric gels, multitask graph neural networks and multimodal encodings capture repeat unit chemistry, topology, and composition to predict properties with limited data, enabling inverse search with reinforcement learning, grammar constrained generators, and Bayesian acquisition. One few-shot framework started from only 86 host defense peptide-mimicking polymers, simulated more than ten to the five possibilities, and identified 83 optimal candidates that were synthesized and validated, illustrating how grammar constraints and reward shaping can keep proposals inside a chemically valid and synthesizable subspace while meeting multiple design criteria [130]. Beyond generators, Bayesian optimization and evolutionary search remain powerful for composition and crosslink density tuning in hydrogel metamaterials and printed lattices, especially when coupled to constraints that enforce extrudability, gel point, and ionic strength windows relevant to biointerfaces [136,139]. These advances show that machine learning can transform hydrogel design into a predictable and efficient search that respects synthesis and processing limits while rapidly delivering biosensing ready materials.

### 4.2. AI Powered Real-Time Physiological Monitoring

Artificial intelligence powered hydrogel biosensors enabled continuous real-time physiological monitoring at the skin interface by combining tissue like soft ionic or electronic conduction with learning-based analytics for pattern discovery and prediction. Recent overviews of wearable chemical and mechanical sensing highlighted continuous monitoring of metabolites and electrolytes in sweat and long duration tracking of biomechanical motion, laying a foundation on which artificial intelligence methods operated to extract clinically relevant features from streaming data [122]. The coupling of compliant hydrogel interfaces with artificial intelligence pipelines supported noninvasive, high fidelity, real-time measurements suitable for personalized health management [140,141].

**Figure 5:**
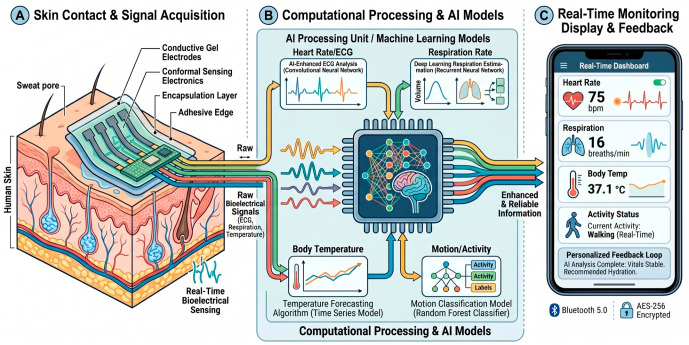
Conceptual architecture and data workflow of an AI-powered real-time physiological monitoring system utilizing conformal conductive hydrogel wearables.

The schematic Figure 5 illustrates the end-to-end integration of soft biomaterials with on-device machine learning analytics for continuous, closed-loop health tracking. At the tissue-device interface, a highly conformal, multi-layered conductive hydrogel patch establishes low-impedance contact with the epidermis, acquiring multimodal raw bioelectrical signals (e.g., electrocardiogram, respiration dynamics, and temperature) while minimizing motion artifacts during physical activity. These raw, correlated time-series data streams are continuously transmitted to a dedicated computational unit where task-specific machine learning models, such as convolutional neural networks for ECG deconvolution, recurrent neural networks for respiration estimation, and time-series forecasting for thermal dynamics, extract clinically relevant features. Ultimately, this processed data is synthesized into a dynamic, user-facing dashboard, delivering predictive health insights, multi-parameter vital sign tracking, and personalized physiological feedback in real time.

From a material and transduction perspective, conductive hydrogels were designed by percolation of electronic fillers or by high-density ionic networks that formed electric double layers at embedded electrodes, which increased interfacial capacitance and reduced contact impedance. Reviews summarized how composition and network architecture governed conductivity, stretchability, adhesion, and water retention that were critical for skin-worn operation [142,143]. Quantitatively, an antifreezing ionic hydrogel achieved an elongation at break of about 3600% and electrical conductivity of 9.82 mS/cm, which corresponded to about 0.982 S/m, while maintaining sensing stability across large deformation [144]. A soft-matter hydrogel with carbon nanotube and ionic charge carriers reached an electrical conductivity of 6.67 S/m with repeatable adhesion on skin, indicating robust ionic electronic coupling under sweat and motion [145]. The key finding was that controlled network design and ion management produced skin-like hydrogels with conductivities near 1 to 10 S/m and extreme stretch without delamination, which are preconditions for stable real-time sensing.

For biomechanical monitoring, hydrogel strain and pressure sensors translated deformation to resistance or capacitance change that followed percolation or tunneling dominated transport models. Gauge factor, defined as the slope of fractional resistance change versus strain, summarized sensitivity while dynamic response quantified temporal resolution. Gelatin-based conductive hydrogels exhibited a gauge factor of about 3.28 with a detection limit of 0.1% strain, supporting subtle motion tracking [146]. Instant self-healing adhesive hydrogels achieved a gauge factor of about 1.27 with a response time of 0.84 s and retained stable output over 500 cycles between 0% and 300% strain [147]. A structural strain concentration design tuned gauge factor continuously from about 1.31 to 9.21 over 0% to 100% strain, demonstrating theory-guided geometry for sensitivity control [148]. Hydrogel fiber sensors maintained function under cyclic stretching at about 8.6 Hz with a response and recovery time of about 30 ms, which matched the bandwidth of gait and respiration monitoring [149]. The key finding was that modern conductive hydrogel sensors provided tunable gauge factor from nearly 1 to about 10 with sub-percent resolution and millisecond response, thereby supporting real-time biomechanics analytics.

Electrochemical hydrogel platforms enabled sweat analysis for electrolytes, metabolites, and small molecules through enzymatic amperometry, potentiometry, optical readout, or surface-enhanced Raman spectroscopy. Reviews described microfluidic sampling, iontophoresis for sweat induction, and multiplexed sensing integrated with wireless modules for real-time readout [150,151]. Recent machine-learning-assisted surface enhanced Raman-spectroscopy-quantified glucose in real sweat, where gradient boosted trees and support vector machine models showed root mean square errors of about 4.93 and 7.52 in the reported concentration units, indicating substantial performance gaps between algorithms for the same spectra [152]. Machine-learning-driven wearable sweat systems simultaneously measured glucose, lactate, urea, and acidity and then fused multichannel data to estimate physiology in real time, illustrating the advantage of feature learning over single-channel thresholds [153]. The key finding was that artificial intelligence transformed noisy, variable sweat signals into quantitative estimates with documented prediction errors at the single-digit level in the reported units, which moved sweat sensing toward actionable physiology.

Skin conformal hydrogel electrodes supported real-time electrophysiology by providing a hydrated ion rich interface that minimized skin electrode impedance and motion induced artifacts. In neural interfaces, hydrogel-based scalp electrodes maintained impedance between about 5 kΩ and 15 kΩ for more than 10 h and supported eight-channel brain–computer interaction without short circuits [154]. Conductive-hydrogel-enabled electrodes delivered ultralow impedance reported as about tenfold lower than silver chloride controls while acquiring stable alpha rhythm electroencephalography, electrocardiography, and electromyography, underscoring the impedance matching advantage of hydrated gels [155]. A thermo-responsive gel electrode decreased skin contact impedance by more than one order of magnitude compared with dry counterparts, which clarified the mechanistic role of interfacial water and ion transport in reducing polarization impedance [156]. Hydrogel electrodes consistently reduced skin contact impedance by factors of about 10 and preserved signal quality in long-duration recording, enabling artificial intelligence models to operate on cleaner biopotentials for robust inference.

Artificial intelligence methods for hydrogel biosensors relied on supervised and self-supervised learning for time series, including convolutional neural networks for spectral and waveform feature extraction, gradient boosted trees for tabular fusion of chemical and physical channels, and sequence models for temporal forecasting. Multiparametric fusion that combined sweat lactate, sweat rate, and heart rate was proposed to estimate blood lactate more accurately than any single channel, reflecting a systems identification viewpoint in which hidden physiological states were inferred from correlated observations [157]. Optical and electrochemical surface-enhanced Raman spectroscopy pipelines benefited from automated baseline correction, peak selection, and nonlinear regression, where algorithm choice altered absolute error by a factor of about 1.5 based on reported root mean square error differences for glucose prediction [158]. Model structure and multimodal fusion strongly governed error bounds; so, theory-guided feature engineering and regularization were as important as sensor hardware in achieving clinically meaningful accuracy.

System design for real-time operation emphasized adhesion mechanics, antifreezing and anti-drying chemistry, and energy autonomy. Antifreezing eutectic or organohydrogel formulations preserved ionic conductivity below 0 °C and reduced evaporation at elevated temperature, which maintained signal stability outdoors [159]. Self-powered lactate biofuel cell integrations and other energy harvesters provided micro-watt to milli-watt scale power that sustained intermittent sensing and Bluetooth transmission during exercise [160]. Material studies reported reusable adhesion stresses around 104 kPa with stable bonding to skin, which limited relative slip and reduced motion artifact under sweat [143]. Chemical strategies for water retention and antifreezing, coupled with low-power electronics and biofuel cell harvesting, enabled practical day long real-time monitoring without failure of the hydrogel interface.

Design theories guided by polymer physics and transport modeling improved robustness. Flory-type network theory and time–temperature superposition explained viscoelastic relaxation and hysteresis that otherwise distorted strain readouts, while percolation theory and tunneling conduction models predicted gauge factor trends with filler volume fraction and crack geometry [161]. In electrochemical sensing, Nernstian potentiometry and Michaelis Menten enzyme kinetics described steady-state calibration, and equivalent circuit analysis of the electrode–skin interface quantified the role of electric double-layer capacitance and charge-transfer resistance in noise shaping [151]. For real-time inference, recursive state estimation such as Kalman filtering and drift compensation stabilized predictions when sweat rate or temperature varied, and cross-calibration to internal references reduced bias. The key finding was that explicit physical and chemical models, coupled with statistical learning, reduced drift and improved generalization beyond a single environment, which is essential for at home continuous use.

Transduction breadth expanded beyond mechanics and electrochemistry to optics integrated with hydrogels. Epidermal optical platforms used colorimetry, fluorescence, electrochemiluminescence, and surface enhanced Raman spectroscopy within hydrogel or hydrogel supported microfluidics to monitor sweat acidity and metabolites with on-body readout. Plasmonic double network hydrogels enhanced Raman scattering to detect urea, uric acid, and lactic acid at trace levels while remaining soft and stretchable, which supported on skin spectroscopy under motion [158]. Breath and cardiorespiratory monitoring also benefited from compliant materials that maintained intimate skin contact for pressure and flow sensing while avoiding occlusion, further enriching multimodal inputs for artificial intelligence fusion [162]. The key finding was that hydrogel compatible optics and soft mechanics widened the data spectrum available to learning algorithms, allowing for cross-checking between modalities to contain error.

Soft hydrogel electrodes provide stable, low-impedance coupling that preserves the microvolt scale dynamics of electroencephalography (EEG), electrocardiography (ECG), and electromyography (EMG) for real-time inference. Semidry hydrogel EEG contacts have maintained skin impedance below 0.4 kΩ for as long as 12 h with event-related potentials comparable to wet paste, while classic dry pins often sit near 100 kΩ in the 0.5 to 50 Hz band and miss induced components [2,13]. Poly(3,4 ethylenedioxythiophene):polystyrene sulfonate (PEDOT:PSS)-based hydrogel interfaces further lower interface impedance and lift signal to noise by about 2.1 dB on average with a maximum of 3.4 dB relative to clinical electrodes, enabling motion robust scalp EEG and surface EMG [2,163]. Stretchable adhesive EMG arrays achieve about 200% strain tolerance, peeling force near 0.58 N cm^−1^, and stable twenty plus channel recordings over more than five days, supporting tendon localization and fatigue tracking during dynamic tasks [164]. Design theory links these gains to increased real contact area and reduced complex impedance Z_skin_ through ionic conduction and mixed electronic transport, which improves the effective signal-to-noise ratio without aggressive skin preparation [165]. For long-wear ECG, breathable hydrogel fabrics and dry blends keep contact impedance below a few kΩ while retaining hydration, which reduces baseline wander and allows shallow models to hit sensitivity and specificity above 95 percent with fewer parameters and lower energy per inference [166]. Microstructured hydrogel contacts further stabilize baselines during sweat and motion and compare favorably to Ag AgCl for both impedance and comfort [167,168].

### 4.3. Enhancing Diagnostic Accuracy Through Multimodal Data Fusion

Hydrogel platforms make it practical to collect complementary signals from the same skin site and to fuse them in algorithms that exploit redundancy and synergy. Typical combinations include bioelectric cues from electroencephalography, electrocardiography, and electromyography together with mechanical strain or pressure and biochemical readouts from sweat or interstitial fluid, all supported by low-impedance hydrogel contacts or conductive polymer hydrogels [24,67,68,169].

**Figure 6:**
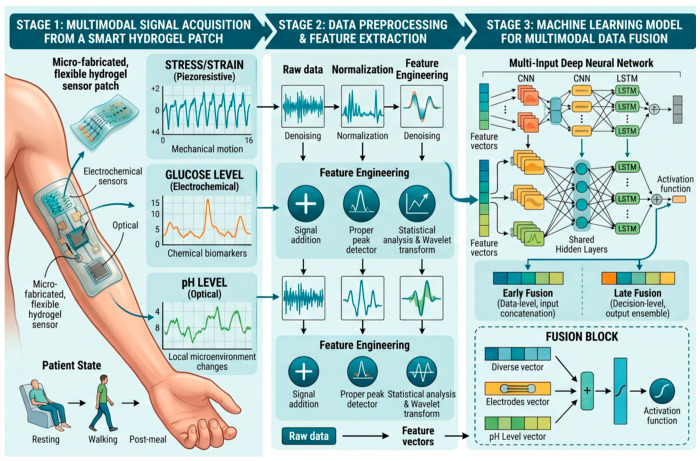
Integrated workflow of a machine-learning-enabled multimodal data fusion system for wearable hydrogel biosensors and enhanced health diagnostics.

Figure 6 illustrates the comprehensive system architecture of a machine-learning-enabled multimodal data fusion approach for wearable hydrogel biosensors, designed to overcome the limitations of single-modal sensing. The workflow progresses through three stages: (Stage 1) multimodal signal acquisition from a smart hydrogel patch, where a flexible hydrogel patch captures diverse real-time mechanical (stress/strain), chemical (glucose level), and optical (pH level) signals under various patient states; (Stage 2) data preprocessing & feature extraction, where raw signals are denoised, normalized, and processed using specific feature engineering methods to yield refined feature vectors; and (Stage 3) machine learning model for multimodal data fusion, which utilizes a multi-input deep neural network (incorporating CNN and LSTM layers) and diverse fusion strategies, including early and late fusion, to integrate the multi-dimensional feature data.

Theory from sensor fusion distinguishes data level fusion that concatenates synchronized waveforms, feature level fusion that aligns latent embeddings through canonical correlation or tensor correlation, and decision-level fusion that ensembles calibrated classifiers, with Bayesian and state-space formulations providing principled uncertainty handling for clinical thresholds [170,171,172]. Across wearable studies, multimodal pipelines consistently outperform single channels. Fused electroencephalography with peripheral physiology via deep encodings reported an accuracy of 90.96 percent and an F1 score of 91.67 percent for stress-state classification, highlighting the benefit of cross-signal structure when trained on thousands of windows [80]. A curated Scientific Data resource with thirty-five participants and synchronized fifty-nine channel electroencephalography, single-channel electrocardiography, four-channel electromyography, skin conductance, and eye movement streams illustrates the scale and alignment quality now available for training and validation of fusion models [173]. Hydrogel-based electronic skins further expand spatial information. Electrical impedance tomography with hydrogel conductors reconstructs contact maps that are then fused with strain and temperature to classify gestures and touch events, a workflow demonstrated in flexible hydrogel electronic skins and in triboelectric impedance tomography wearables that image tissue with low drive and real-time frames [24,169]. Material advances that matter for fusion include sandwich polyvinyl alcohol and poly(3,4 ethylenedioxythiophene):polystyrene sulfonate electrodes that lower skin interface impedance while maintaining stretch, ionic double networks that keep conductivity under large deformation, and hybrid liquid metal hydrogels that sustain working strain up to two thousand three hundred percent, all of which reduce motion artifacts and improve cross-modal alignment before learning [174,175,176]. Reviews across hydrogel wearables, stretchable hydrogels, and multimodal skin like sensors converge on the same design message. Stable adhesion, water retention, and matched modulus improve correlation across modalities and therefore tighten the confidence bounds that Bayesian fusion reports at inference time [63,177,178].

In practice, flexible hydrogel patches have combined proximity, pressure, and temperature sensing to monitor breathing and sleep without wires, while feature level fusion improved respiratory phase segmentation relative to single channels on multi night recordings [63]. Sweat analytics adds orthogonal information. Hydrogel microfluidics capture lactate, glucose, pH, sodium, and cortisol together with heart rhythm and motion, enabling joint models that reduce false positives in exertion and illness screening compared to electrocardiography alone [150,176]. Broader wearable fusion surveys report typical gains of several points in accuracy or area under the curve and improved calibration when probability outputs are temperature scaled and aggregated with Bayesian rules, with latency suitable for on-body or near-body processors [171,172]. Materials for reliable interfaces matter here as well. Electrode structure and biocompatibility as primary levers for low impedance and hydration stability, reduce missing data and lower alignment error during fusion [165,179]. Case studies show ED that hydrogel-based multimodal patches could sustain high stretch while maintaining fidelity for combined strain, temperature, and optoelectronic channels, and that feature fusion improves classification of posture, gesture, and thermal events relative to single channel baselines with modest model sizes that meet edge power budgets [180,181]. Together, these advances show that hydrogel material engineering and principled multimodal fusion raise diagnostic accuracy while keeping models compact and interpretable for real-time use at the skin interface [174,180,182].

## 5. Challenges and Future Directions

Hydrogel biosensors face persistent material and interface challenges that directly affect data fidelity and model robustness. Water loss increases interfacial impedance and baseline drift under ambient wear, while subzero environments can freeze aqueous phases and reduce ionic mobility. Recent organohydrogel chemistries mitigate drying and freezing with water–glycerol or salt-rich matrices that preserve conductivity at temperatures down to about −50 °C while maintaining transparency of about 85% and ionic conductivity around 10–25 mS/cm [183,184,185,186,187]. Tough adhesive hydrogels now reach interfacial toughness near one thousand joules per square meter on wet tissue and adhesion strength around 150 kPa, enabling stable contacts during sweat and motion without skin irritation [188,189,190]. Yet, measured skin–electrode impedance remains highly variable across users and frequency. State-of-the-art paintable biogels have reported interface impedance near 155 Ω at 100 Hz, and dry high-density electrodes benchmarked against gelled Ag/AgCl show comparable or better electroencephalography signal quality when properly designed and preloaded, but with wide subject-to-subject dispersion at 1 Hz to 1 MHz [114,191,192,193]. To translate these material gains into reliable analytics, designs should target modulus matching between device and skin in the 5–200 kPa range, maximize conformal contact to reduce micro-slip, and incorporate uncertainty-aware signal processing such as adaptive filtering and Bayesian state estimation to track time-varying contact impedance and artifact statistics in real use [114].

Algorithmic and data-centric limitations are equally central. Wearable biosignal corpora remain modest compared with imaging, and they are heterogeneous across devices, sites, and populations. A landmark deep learning system trained on 91,232 single-lead electrocardiography traces reached area under the receiver operating characteristic of about 0.97 across 12 rhythm classes, but deployment error still shifted with context and sensor conditions [79]. Open physiological datasets illustrate the scale gap: extended Temple University Hospital electroencephalography now provides tens of thousands of clinical recordings curated for machine learning, while classic MIT-BIH corpora cover 48 records with about 100,000 annotated beats, which is sufficient for benchmarking but not for generalization across demographics and hydrogel interfaces [112,194]. To reduce domain shift between benchtop and on-body data, future pipelines should combine physics-informed data augmentation that explicitly perturbs contact impedance and motion artifact spectra, domain adaptation across sites and chemistries, and calibration methods that report well-calibrated probabilities and epistemic uncertainty for downstream clinical use. Uncertainty quantification can gate closed-loop actions when credible intervals widen due to sweat-induced impedance spikes or loss of conformal contact, reducing false alarms and preventing unsafe actuation.

Power and system integration remain difficult for fully untethered operation, especially when continuous sampling and on-device inference are required. Organic electrochemical transistor front ends operate below one volt and provide high transconductance at the ion–electron interface, which reduces analog front-end overhead and enables in-sensor computing to prefilter and compress signals before radio transmission [195,196,197]. Printed and stretchable organic electrochemical transistor arrays with feature sizes near 100 µm and yields >95% have been demonstrated with coin-sized readout modules, supporting edge classification of biosignals with lower energy per inference than cloud streaming [198,199]. To sustain continuous monitoring, hybrid energy harvesting is promising. State-of-the-art triboelectric nanogenerators report peak instantaneous power densities in the order of hundreds of mW/cm^2^ under ideal mechanical drive, while recent epidermal lactate biofuel cells provide about 1–2 mW/cm^2^ in artificial sweat at 20 mM lactate, enough to offset sensing and inference duty cycles in short bursts [200,201,202,203]. In practice, harvested power is intermittent and user-specific. Designs should therefore pair energy-aware inference schedules with event-triggered sensing, reservoir computing, and low-rank model compression to keep average power below one milliwatt while preserving clinical sensitivity [203].

Biofouling, corrosion, and long-term biostability constrain multiweek wear or implantation. Zwitterionic and other hydration-layer forming coatings can cut nonspecific protein adsorption to <5 ng/cm^2^ in serum and reduce platelet and cell adhesion during hours-long tests, which stabilizes baselines and prolongs calibration intervals [204,205,206]. Photografted zwitterionic thin films on elastomers reduced fibrinogen adsorption by about twenty fold while retaining elasticity, offering a path to antifouling skins for hydrogel electrodes that must stay soft. Future work should combine such nonfouling skins with ionically conductive, antifreezing organohydrogels so that adhesion, hydration, and charge transport are co-optimized rather than traded-off.

**Figure 7:**
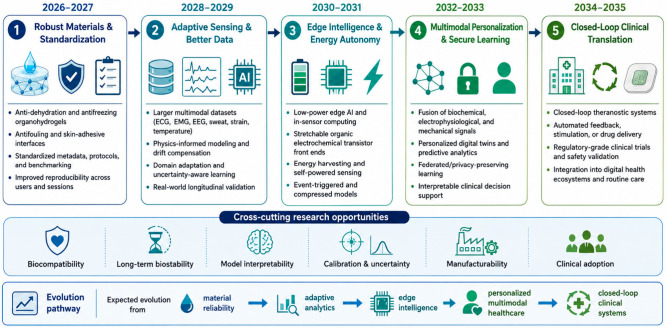
Ten-year roadmap of future research opportunities for machine-learning-enabled hydrogel biosensors in wearable health monitoring.

Figure 7 presents a 10-year roadmap outlining the expected evolution and key research opportunities for machine-learning-enabled hydrogel biosensors in wearable health monitoring. In the near term, from 2026 to 2027, progress is expected to be driven by robust materials and standardization, including anti-dehydration and antifreezing organohydrogels, antifouling and skin-adhesive interfaces, standardized metadata reporting, and reproducible benchmarking across users, sessions, and environments. During 2028–2029, the field is likely to shift toward adaptive sensing and higher-quality datasets, where larger multimodal datasets, physics-informed modeling, drift compensation, uncertainty-aware learning, and longitudinal real-world validation will become central. From 2030 to 2031, research priorities are expected to move toward edge intelligence and energy autonomy, including low-power edge AI, in-sensor computing, stretchable organic electrochemical transistor front ends, energy harvesting, and event-triggered compressed models for continuous on-body operation. In the 2032–2033 period, multimodal personalization and secure learning will become increasingly important, with opportunities in biochemical–electrophysiological–mechanical signal fusion, personalized digital twins, federated learning, privacy-preserving model training, and interpretable clinical decision support. Looking further ahead to 2034–2035, hydrogel biosensors may evolve into closed-loop clinical systems that integrate continuous sensing with automated feedback, stimulation, or drug delivery, supported by regulatory-grade clinical trials and digital health infrastructure. Across all stages, biocompatibility, long-term biostability, model interpretability, calibration and uncertainty quantification, manufacturability, and clinical adoption will remain cross-cutting challenges. This roadmap highlights that the future of hydrogel biosensors will not depend on isolated improvements in materials, devices, or algorithms alone, but rather on coordinated co-design across soft material chemistry, biointerface engineering, low-power electronics, machine learning, clinical validation, and data governance.

Among these long-term opportunities, closed-loop theranostic systems represent a particularly important direction, as they pair hydrogel biosensors with on-board stimulation or drug delivery under machine learning control. In neuroscience, wearable and implant-adjacent platforms already demonstrate bidirectional recording and stimulation in mobile humans, along with reinforcement learning and model-based controllers for deep brain stimulation that adapt parameters to biomarkers in real time, which reduces energy use and improves symptom control relative to manual programming [207,208,209,210]. Translating that blueprint to hydrogel biosensing suggests controllers that pause or attenuate actuation whenever uncertainty spikes, that recalibrate using active learning when drift exceeds preset thresholds, and that rely on causal feature selection rather than purely correlational patterns to prevent spurious feedback. Measurable targets for the next five years include maintaining interface impedance within two times of initial values over seventy-two-hour wear without rehydration, limiting prediction error growth under domain shift to less than 5% after 24 h of unsupervised adaptation, and sustaining average power budgets below one milliwatt through in-sensor computing and energy harvesting [201,202]. Achieving these targets will require co-design across hydrogel chemistry, device physics, signal processing, and machine learning, plus rigorous adoption of reporting standards and privacy-preserving training so that the results generalize across people, places, and products [211,212].

## Figures and Tables

**Table 1:** Summary of hydrogel material property datasets and descriptors.

Dataset Name	Dataset Size and Context	Descriptors	Ref
Hydrogel printability database	1568 assays; ten hydrogels; bioprinting	16 properties; viscosity (Pa·s), G′/G″ (Pa), G′ (storage modulus): elastic stiffness. Energy stored per cycle. G″ (loss modulus): viscous resistance. Energy dissipated as heat.	[36]
Hydrogel characterization: GelMA & PEGDA	Two hydrogels; mechanical, ultrasound, rheology	Young’s modulus (Pa); viscosity (Pa·s); density (kg/m^3^)	[37]
Nucleoside hydrogel-forming dataset	71 derivatives; gelator/non-gelator labels	4175 molecular descriptors; binary gelation; dimensionless	[38]
Hydrogel rheology-printability (DIW) database	150 hydrogels; DIW; printability modeling	G′/G″ (Pa); viscosity (Pa·s); yield stress (Pa)	[39]
Underwater adhesive hydrogel design dataset	180 bioinspired hydrogels; underwater adhesion	Monomer fractions; adhesion F_a_ (MPa); G′ (Pa);	[40]
Dipeptide hydrogel library	>2000 peptides; gelation screening	Physicochemical descriptors; gelation outcome; dimensionless	[41]
Polyacrylamide hydrogel characterization dataset	Formulations; rheology, UV-vis, swelling	G′/G″ (Pa); absorbance; swelling ratio	[42]

**Table 2:** Summary of major sensing mechanisms in hydrogel-based biosensors.

Mechanism	Hydrogel Type	Target Analyte/Parameter	Signal Output	Biosensor Performance	Representative Application
Electrochemical	Enzyme-functional conductive hydrogel	Glucose, lactate	Current, voltage, impedance	High sensitivity; fast response	Sweat glucose, lactate, ions [57,58]
Optical	Plasmonic hydrogel substrate	Lactate, urea, uric acid	Fluorescence, color, wavelength shift	Trace-level molecular detection	Sweat pH, optical metabolites [59]
Mechanical	Conductive stretchable hydrogel	Strain, pressure	Resistance, capacitance, frequency	Wide strain range; repeatable cycles	Motion, respiration, pulse [60,61]
Multimodal	Hydrogel electronic skin	Strain, temperature, touch	Fused multimodal signals	Gesture and touch classification	Sleep, HMI, health tracking [62,63]

**Table 3:** Machine learning models and reported biosensor performance.

Biosensor/Target	Input Signal	ML Model	Reported Performance	Analytical Gap	Ref.
Single-lead ECG arrhythmia	ECG waveform	Deep neural network	AUROC 0.97	Hydrogel–interface shift untested	[79]
Wearable stress sensing	EDA, ACC, BVP, temperature	Image-transformed deep learning	Accuracy 90.96%; F1 91.67%	Free-living validation limited	[80]
Hybrid EMG–FMG gestures	EMG and FMG features	SVM, kNN, LDA	Accuracy 99.07%; gauze 99.42%	Long-wear robustness unclear	[81]
Co-located EMG–FMG armband	EMG and FMG features	Gesture classifier	EMG 81.5%; FMG 80.6%; fused 91.6%	Subject diversity limited	[82]
Nucleoside hydrogel prediction	Molecular descriptors	Logistic regression	Accuracy 71%; external 83.3%	Small dataset; interpretability limited	[38]
Granular alginate hydrogel design	Composition and process variables	Supervised ensemble pipeline	Accuracy 0.885; AUC 0.955	Signal–performance coupling missing	[45]

**Table 4:** Algorithmic comparison for hydrogel biosensor analysis.

ML Algorithm	Suitable Biosensor Data	Performance Strength	Robustness Concern	Best Applicability
SVM, RF, GBDT	Engineered tabular features	Strong small-data performance	Feature quality dependent	EIS, voltammetry, sweat analysis [7]
CNNs	Raw waveforms or spectra	Automatic feature extraction	Needs larger datasets	ECG, EEG, EMG sensing [124]
RNNs, LSTMs	Sequential wearable signals	Temporal pattern learning	Domain shift sensitive	Respiration, motion, biosignal streams [8]
Transformers	Long multimodal time series	Long-range dependency learning	Data and compute intensive	Multimodal health monitoring [125]
Bayesian optimization	Formulation or printing variables	Efficient experimental search	Prior and kernel sensitive	Hydrogel ink optimization [45,126]
GNNs	Polymer molecular structures	Structure–property prediction	Limited labeled polymers	Hydrogel material informatics [127]
Self-supervised models	Unlabeled polymer datasets	Data-efficient representation learning	Pretraining quality dependent	Low-data formulation screening [20]
Physics-informed models	EIS, transport, mechanics	Improved physical consistency	Requires valid physics	Drift correction, parameter fitting [128]
Transfer learning	Cross-user wearable data	Reduces recalibration burden	Negative transfer risk	Personalized wearable deployment [129]
Multimodal fusion	Synchronized multisensor signals	Richer diagnostic information	Crosstalk and alignment errors	Sweat, strain, ECG fusion [71,125,130]

## Data Availability

No new data were created or analyzed in this study. Data sharing is not applicable to this article.
